# DISTINGUISHED FACULTY AND RISING STAR EARLY-CAREER FACULTY AWARD LECTURES

**DOI:** 10.1093/geroni/igz038.1400

**Published:** 2019-11-08

**Authors:** Cynthia Hancock

**Affiliations:** University of North Carolina, Charlotte, Charlotte, North Carolina, United States

## Abstract

The Distinguished Faculty Award acknowledges individuals whose teaching stands out as exemplary, innovative, or influential—or any combination thereof. The Distinguished Faculty Award lecture will feature an address by 2019 recipient Gayle Doll, PhD, of Kansas State University. The Rising Star Early-Career Faculty Award acknowledges new faculty whose teaching and leadership stand out as influential and innovative. The Rising Star Early-Career Faculty Award lecture will feature an address by 2019 recipient Katarina Felstedof, PhD, of the University of Utah.

